# Innovations in pancreatic anastomosis technique during pancreatoduodenectomies

**DOI:** 10.1007/s00423-020-01942-8

**Published:** 2020-07-31

**Authors:** S. Ferencz, Zs. Bíró, A. Vereczkei, D. Kelemen

**Affiliations:** 1 Department of Surgery, Clinical Center, Medical School, University of Pécs, Pécs, Hungary; 2 Division of Surgery, County Hospital, Szombathely, Hungary

**Keywords:** Pancreatic resection, Purse-string suture pancreatojejunostomy Pancreatic fistula, Drain amylase level

## Abstract

**Purpose:**

Pancreatic fistula following pancreatic resections is still a relevant complication. The present work shows the efforts of a single institute to decrease this problem.

**Methods:**

A total of 130 patients (63 men, 67 women) with a mean age of 60 (range: 23–81) years were operated on between January 2013 and March 2020. The most frequent type of pancreatic resection was a Whipple procedure with partial antrectomy. During all operations, an innovative method was used, namely a modification of the purse-string suture pancreatojejunostomy. Moreover, an early drain removal policy was applied, based on the drain amylase level on the first and subsequent postoperative days.

**Results:**

Mean postoperative hospital stay was 13 days (range: 7–75). The overall morbidity rate was 43.8%; the clinically relevant (grade B/C) pancreatic fistula (CR-POPF) rate was 6.9%. Delayed gastric emptying (DGE) was observed in 4% of the patients. The ratio of operative mortality was 0.7%; the reoperation rate was 5.3%. Based on the drain amylase level on the first postoperative day, two groups could be established. In the first one, the drain was removed early, on the fourth day in average (range: 2–6). In the other group, the drain was left in situ protractedly or reinserted later on.

**Conclusion:**

A single center’s experience proves that the refinement of the technique can improve the results of pancreatic surgery.

## Introduction

Surgical morbidity rate after pancreatic resections is still high (up to 50%) even in specialized centers. Beside delayed gastric emptying, biliary fistula, postoperative hemorrhage, surgical site infection, and other morbidities, pancreatic fistula is the most relevant complication with a rate of 10–15% after pancreatoduodenectomies [1]. Many efforts have been done to decrease this number, like several modifications of the pancreato-enteric anastomosis, stenting of the pancreatic duct, administration of somatostatin, etc.; however, no single method has been proven to be superior, according to the reviews and meta-analyses [2]. That is why pancreatic surgeons have continuously tried to find the ideal method for decades. The present paper shows such efforts of a single institute.

## Material and methods

**Fig. 1:**
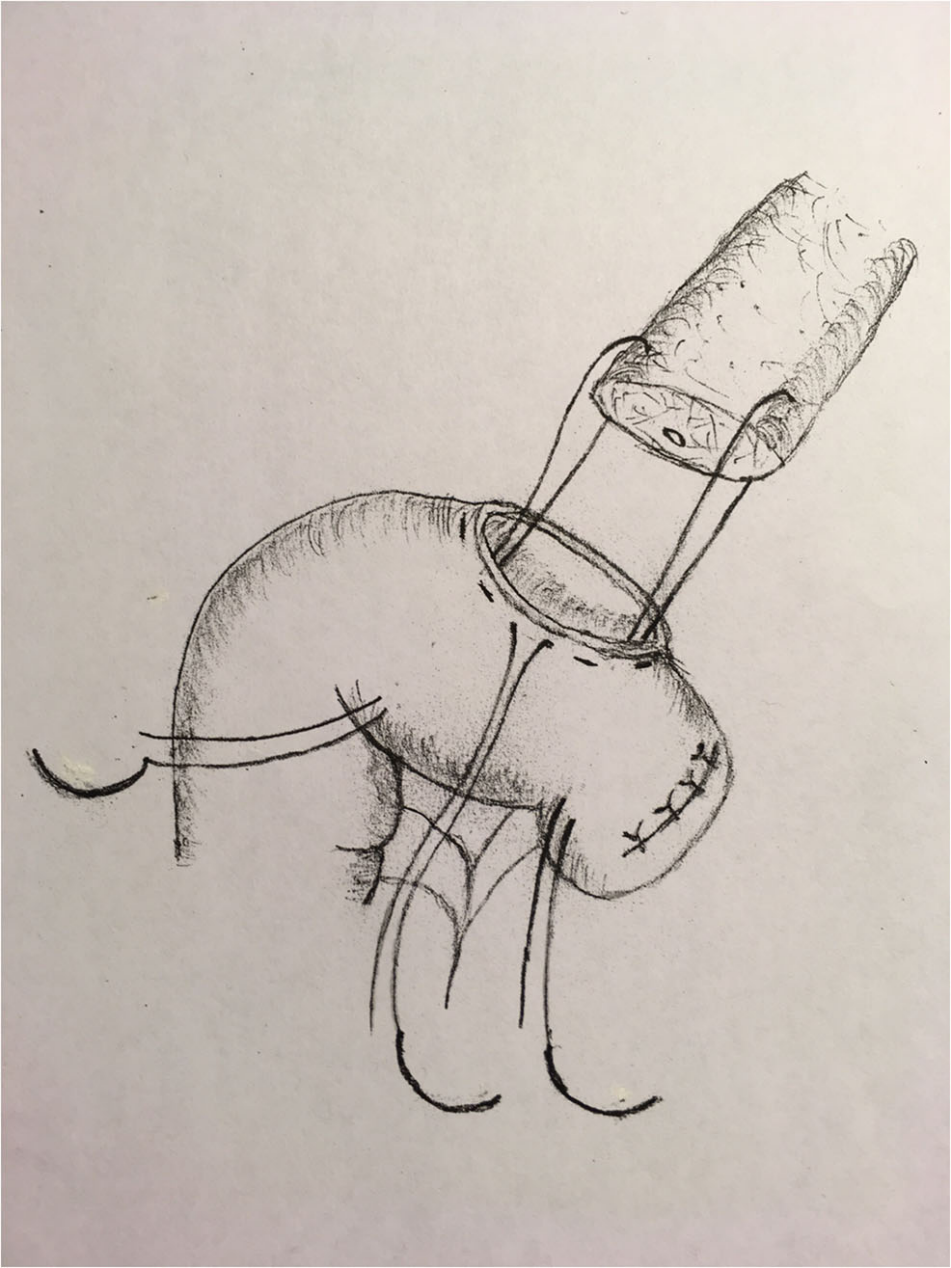
Schematic drawing of the end-to-side pancreatojejunostomy, created with a single purse-string stitch and two U-shaped fixing sutures

**Fig. 2:**
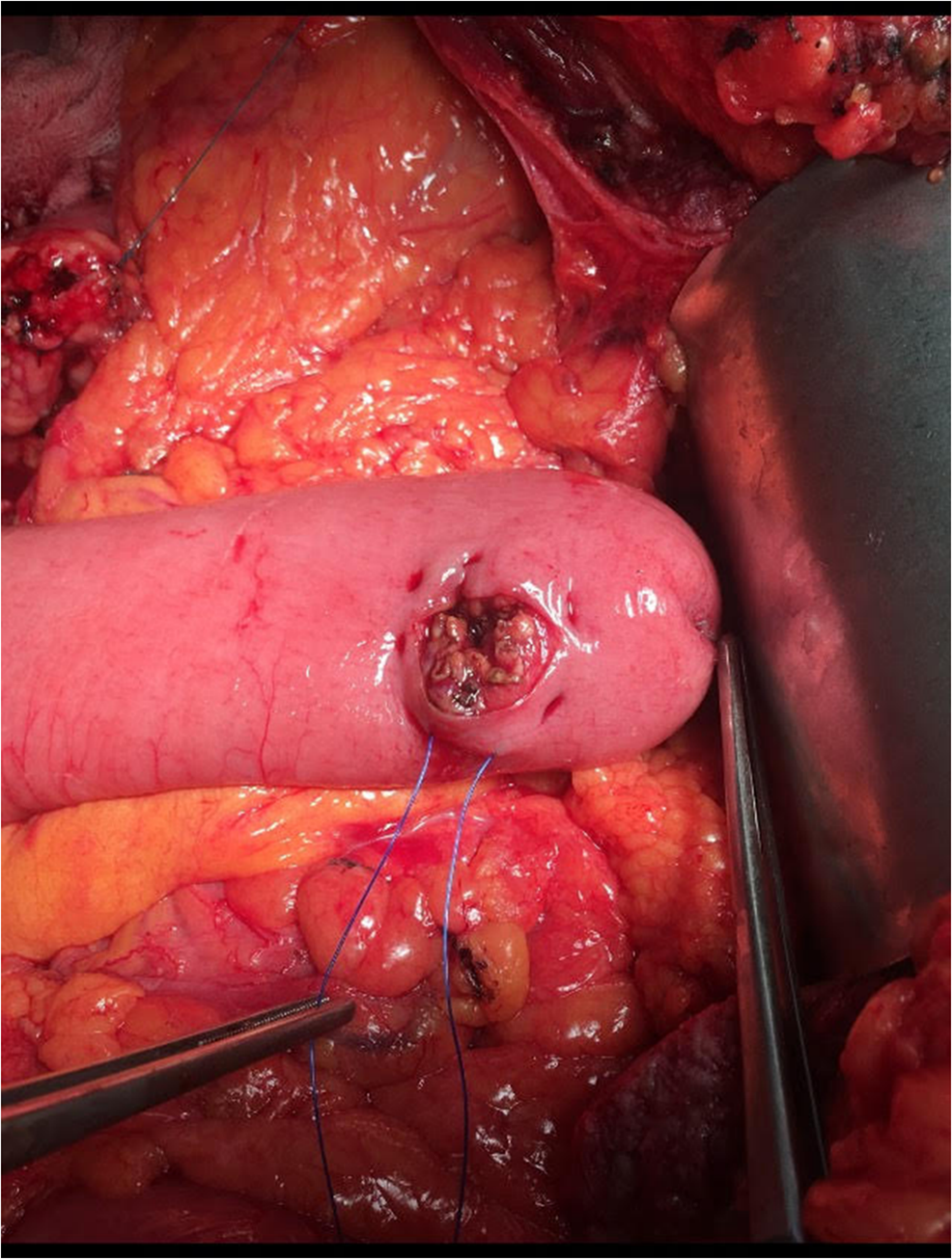
A purse-string suture was put around the jejunal opening

**Fig. 3:**
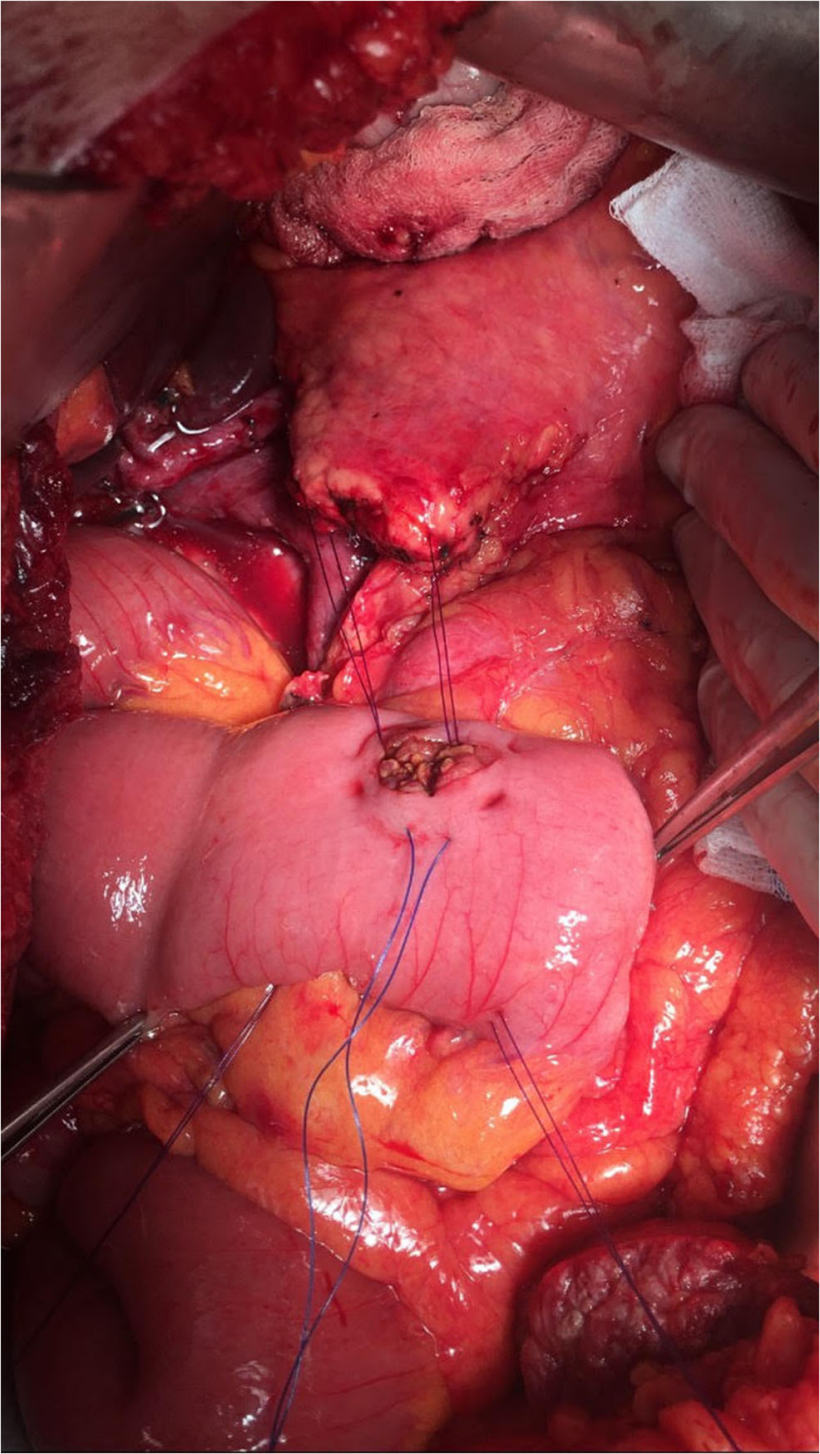
Two U-shaped fixing sutures were placed

**Fig. 4:**
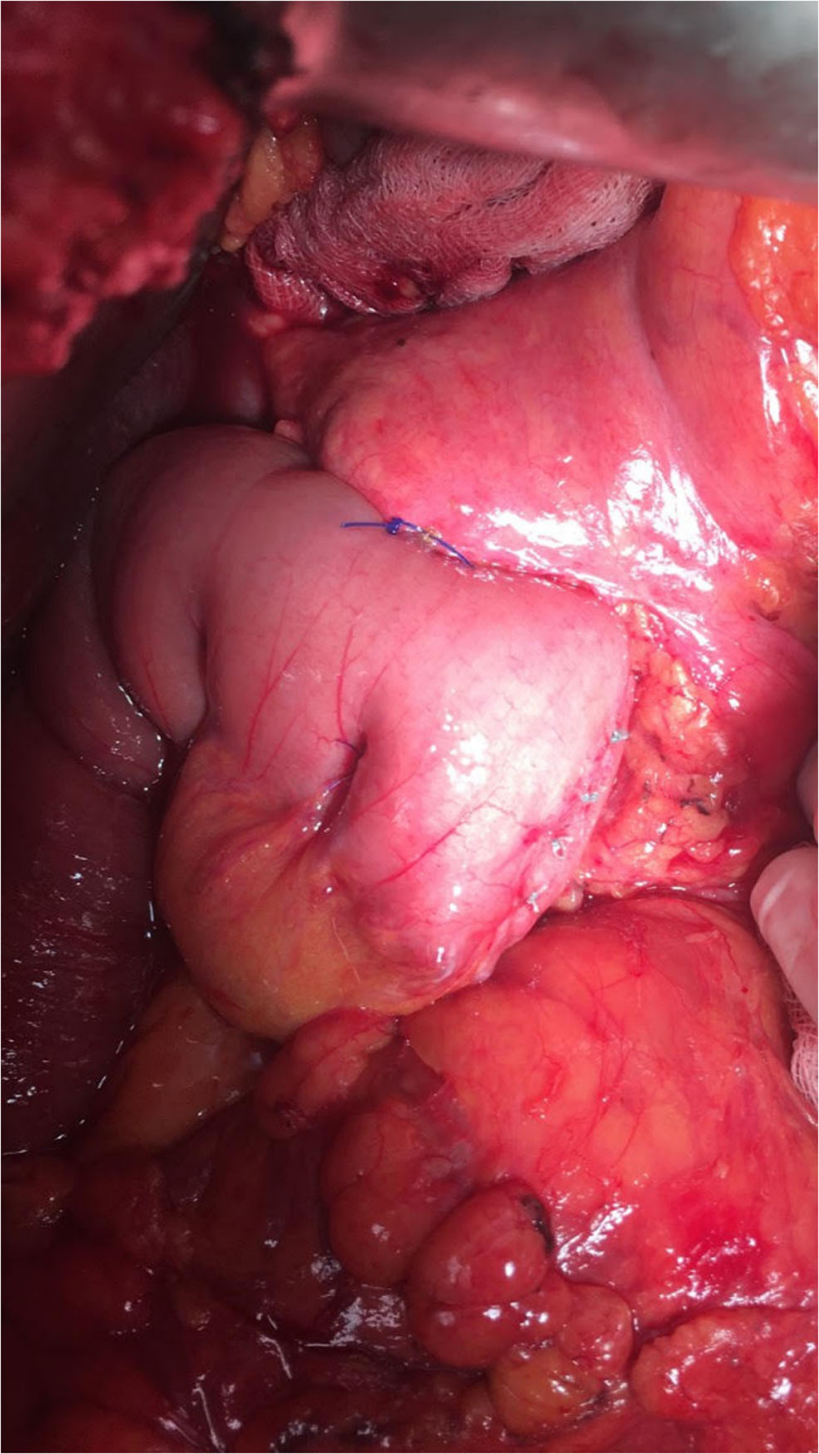
The ready anastomosis after knotting of the three stitches

Between January 2013 and March 2020, 130 Whipple procedures (74 with partial antrectomy and 56 with preservation of the pylorus) were performed at the Department of Surgery, Clinical Center, Medical School, University of Pécs, Pécs, Hungary. Table 1 summarizes the patients’ data. The gender distribution was almost equal and the mean age was 60 years. The most common disease was a pancreatic neoplasia. During the procedure—after the radical resectional phase—a very simple type end-to-side pancreatojejunostomy was created with only three stitches. After mobilization of the pancreatic stump up to 2–3 cm distally, on the antimesenteric border of the jejunal limb, an enterotomy was made with a length of 2/3rd the diameter of the stump in order to get a tight contact after the implantation of the pancreas into the bowel lumen. Afterwards, a seromuscular 2/0 monofilament nonabsorbable purse-string suture was put in the bowel wall about 3–4 mm from the edge of the opening. The next step was to put two U-shaped fixing sutures to the cranial and caudal corner of the pancreas (order: jejunum outside-in—pancreatic corner—jejunum inside-out), as well, with 3/0 monofilament absorbable suture material. Care was taken not to hurt the small vessels at the mesenteric border with the U stitches (Figs. 1, 2, 3, and 4.) By knotting the U stitches, the pancreas was implanted and fixed into the bowel; then, the purse-string suture was knotted. Our first experiences with this technique were previously published [3, 4]. Noteworthy tricks during the creation of the anastomosis were identified, namely the importance of turning the jejunal mucosa into the bowel lumen with a fine dissector Pean before tightening the purse-string stitch. Thus, the bowel serosa touched to the pancreatic surface, which is a prerequisite for the healing of the anastomosis. Avoidance of supplementary stitches is important, as the essence lies in the application of a single suture. The tightness of the knot was gently checked with a metal probe. Moreover, the knot of the U stitches was covered with a single serosal suture. Our technique is a simple modification of the purse-string suture pancreato-enteric anastomosis, which was first published by Spivack and Wile [5], then popularized by others [6]. One soft silicon drain was positioned in front of the pancreatojejunostomy and this area was covered with the omentum in order to fix the drain and also to create a localized space for the case of pancreatic fistula. The drain was placed after the operating table was put back to the flat position. The number of cases with normal parenchymal texture (66) was almost equal to the fibrotic one (64). The order of the further anastomoses was hepaticojejunostomy (continuous suture in case of a dilated duct and interrupted stitches in case of a narrow one), then antecolic duodeno-, or gastrojejunostomy with an additional Braun anastomosis between the afferent and efferent loop. During the operation, regional lymphadenectomy was routinely performed. In the perioperative period, the enhanced recovery principles were applied, like preoperative counseling, avoidance of preoperative biliary drainage (if possible), smoking and alcohol cessation, preoperative nutrition (if it needs), chemical and mechanical thromboprophylaxis, antibioprophylaxis and skin preparation, epidural analgesia, avoidance of hypothermia and also hyperglycemia, near-zero fluid balance, early perianastomotic drain removal, omitting somatostatin analogues, stimulation of bowel movement, early enteral feeding, and mobilization, etc. [7]. Drain amylase level was routinely measured on the first postoperative day and also before drain removal. Our aim was to investigate its changes in case of CR-POPF and in the lack of it. The drain management was guided by the policy of the Verona group [8]. The rate of CR-POPF and other complications was also recorded [9, 10]. Octreotide was administered for 7–10 days only in case of a manifest pancreatic fistula.

**Table 1:** Patient data (*n*: 130)

Gender	Male: 63	Female: 67
Mean age	60 years (range: 23–81)	
Diagnosis	Pancreatic neoplasia: 81	
	Neoplasia of the papilla Vateri: 24	
	Distal bile duct neoplasia: 12	
	Chronic pancreatitis: 5	
	Duodenal neoplasia: 4	
	Cystic neoplasia: 4

## Results

Mean postoperative hospital stay (including 2–3 days in ICU) was 13 days (range: 7–75). Tables 2 and 3. show the postoperative results. The overall morbidity rate was 43.8%, the CR-POPF rate was 6.9%, and DGE was in 4% of the cases (grade A). Operative mortality was 0.7% and the reoperation rate was 5.3%. Data of drain amylase level on the first postoperative day was available in about 2/3rd of the cases. Using these numbers, two groups were established (group 1: no fistula, group 2: CR-POPF), presented in Table 4. In group 1, the mean amylase level of drain fluids (2137 U/l, range: 6–46,000) was under 5000 U/l; however, in the group 2, these numbers were much higher (19,550 U/l, range: 28–63,690), except 9 cases in group 1 and one case of group 2, where the level was above or under the 5000 U/l limit, respectively. In group 1, the drain was removed on the fourth day in average (range: 2–6) and on that day the mean amylase level was 264 U/l (range: 3–3370). In group 2, the drain was left in situ or reinserted later on. If there was no recorded data on the first postoperative day, then the time of drain removal was decided according to the visual estimation and/or subsequent determination of drain amylase level. In group 2, two reoperations were needed to perform due to an unsuccessful radiologic drainage of intra-abdominal abscess.

**Table 2:** Nonsurgical morbidity, reoperation, and operative mortality

Nonsurgical complications *n*: 26 (20%)	Pneumonia, respiratory insufficiency, atrial fibrillation, hydrothorax, renal failure, uroinfection
Reoperation *n*: 7 (5.3%)	abdominal wall disruption: 2
	Drainage of intra-abdominal abscess: 2
	Completion pancreatectomy: 1
	Stenosis of hepaticojejunostomy: 1
	Bleeding from the pancreatic resectional surface: 1
Operative mortality *n*: 1 (0.7%)	Due to nonsurgical reason

**Table 3:** Surgical complications *n*: 31, rate: 23.8%

Rate of CR-POPF (B/C)	6.9%	
*n*: 9 (6/3)	In case of soft pancreas (7 out of 66)	10.6%
	In case of fibrotic pancreas (2 out of 64)	3.1%
DGE *n*: 5	4% (grade A)	
Biliary fistula *n*: 0	0%	
Postoperative bleeding *n*: 1	0.76%	
Abdominal wall disruption *n*: 2	1.52%	
Stenosis of hepaticojejunostomy *n*: 1	0.76%	
Wound healing disorder *n*: 14	10.7%

**Table 4:** Groups defined by the mean level of drain amylase on the first postoperative day

Group 1	No fistula (*n*: 75)	2137 U/l (range: 6–46,000), 9 samples above 5000 U/l
Group 2	CR-POPF (*n*: 9)	19,550 U/l (range: 28–63,690) 1 sample under 5000 U/l

## Discussion

Though pancreatic resections are associated with operative mortality in less than 5% of the patients, the morbidity rate is still considerable even in high-volume centers. The greatest problem for the surgeons is still the development of CR-POPF and its consequences, like abscess, hemorrhage, sepsis, multiorgan failure and even death. The huge number of technical innovations and recommendations indicate that pancreatic surgeons have aimed to minimize this problem; however, there has been no general agreement about the ideal method for the prevention [2]. The present work shows the efforts of a single institute. Operative mortality and morbidity rate were 0.7% and 43.8%, respectively. These numbers are similar to the data of other high-volume centers. However, reoperation rate (5.3%) would be lower, if the ultrasound-guided percutaneous drainage of intra-abdominal abscess would have been more successful. Due to the low rate of DGE, the antecolic duodeno-, or gastrojejunostomy with an additional Braun enteroenterostomy was our preferred reconstruction method, similar to others [11]. CR-POPF developed in less than 7% of the patients after Whipple procedure and this number seems to be advantageous, regarding the corresponding data of the literature and also the comparison to the results of our former series with an end-to-side single-layer pancreatojejunostomy. In the latter case the pre- and intraoperative data of 168 patients (age, gender, type of the disease, texture of the pancreas, type of operation) were identical with the present ones. The operative mortality rate was 3.8% (contrary 0.7% in the present series). However, we were not satisfied with the rate of CR-POPF in case of soft pancreas (19%), so our technique was changed to the purse-string suture pancreatojejunostomy, which was associated with a 10.6% fistula rate. The advantage of the latter method may be due to the purse-string suture, when the stitch holes are inside of the bowel lumen. In case of an outside location of the stitch hole, the needle can hurt small pancreatic ducts, generating leakage of pancreatic juice [6]. So the purse-string type suture might be theoretically the key element of the effectiveness of the method. Since its first publication by Spivack and Wile [5], this principle was adopted in several modifications of the technique, for example the report of Nordback, Peng, Bartsch, Hashimoto, Kostov, and Hsu [6, 12–16]. It is important to emphasize that none of these authors applied additional sutures, which resulted in a pancreatic stitch hole outside the bowel lumen. The present series of more than 100 cases shows that the technique is very simple (only three stitches), safe, spares time and also suture material. Kostov and co-workers published the most simple method, namely they used only one purse-string stitch during pancreatogastrostomy, however without any fixing suture.

Recently, there has been a great debate about the use of drainage, either omitting it, or selective drainage, or early removal [8, 17, 18]. Our drain removal policy was basically guided by the 5000 U/l cut-off level of drain amylase [8]; however, the time of drain removal was determined lastly by the current level. According to the absence or presence of CR-POPF, two categories could be distinguished. In group 1, the drain amylase level on the first postoperative day was 2137 U/l in average, so the drain was removed on the mean fourth day postoperatively (at that time, the drain amylase level was 264 U/l in average) and CR-POPF did not develop. However, in group 2 (CR-POPF), the mean amylase level was found to be much higher, 19,550 U/l on the first postoperative day. In these latter instances, the quality of drain effluent was also visually suspicious for fistula and the drain was left in situ. Thus, the drain amylase level on the first day raises the likelihood of fistula development, except nine cases in group 1, and one case in group 2, where the level was above or under the 5000 U/l limit, respectively. In the nine exceptions of group 1, the high amylase level significantly decreased on the subsequent days (no fistula), and in the one exception of group 2, the low amylase level considerably increased later (fistula). It means that drain amylase level on the first postoperative day together with its change and tendency are the dominant factor, whether CR-POPF would develop or not. So before drain removal, it is useful to repeat the measurement. This policy is similar to a recommendation, namely in patients with less than 5000 U/l drain amylase level on the first postoperative day and less than 350 U/l on the third day could be a practical guide for safe early drain removal [19]. Summarizing the drain management, we think that one soft silicon drain (only close to, but not in contact with the anastomosis) for 3–4 days is not able to cause a major problem. However, it gives the opportunity to check the drain amylase level on the first and subsequent postoperative days. As an indicator, it helps us to decide the time of drain removal as early as possible. Without drainage, there is an uncertainty, whether the radiologist will be able to put a drain into a peripancreatic fluid collection in necessity. In case of failure, a reoperation has to be carried out, as in two of our cases. A recently published argument against drainage is that intra-abdominal drains can be dislocated during the postoperative period [20]. We routinely applied two measures to prevent the dislocation, namely the drain was placed after repositioning of the operating table and the pancreatojejunal anastomosis was covered with omentum. Proper position of the drain was detected on CT picture, selectively made in the early postoperative period.

Recently, the so-called “TRIANGLE operation” has been advocated to reach the maximal clearance of tissues between the mesenteric vessels and coeliac trunk during pancreatic cancer surgery [21]. Our first experiences are advantageous also with this technique. As in oncologic surgery generally, radicality and safety of the procedures have paramount importance in pancreatic surgery, too [22, 23].

## Conclusion

Summing up, pancreatic surgeons must refine their own technique to decrease the complication rate as much as possible. The present single institute experience also reflects this ambition, namely the modification of the pancreatic anastomosis technique resulted in a simple and safe method.
